# Genetic Variation in the Interleukin-28B Gene Is Associated with Spontaneous Clearance and Progression of Hepatitis C Virus in Moroccan Patients

**DOI:** 10.1371/journal.pone.0054793

**Published:** 2013-01-24

**Authors:** Sayeh Ezzikouri, Rhimou Alaoui, Khadija Rebbani, Ikram Brahim, Fatima-Zohra Fakhir, Salwa Nadir, Helmut Diepolder, Salim I. Khakoo, Mark Thursz, Soumaya Benjelloun

**Affiliations:** 1 Virology Unit, Viral Hepatitis Laboratory, Pasteur Institute of Morocco, Casablanca, Morocco; 2 Service de Médecine B, CHU Ibn Rochd, Casablanca, Morocco; 3 Ludwig-Maximilians-Universität München, Marchioninistrasse, München, Germany; 4 University of Southampton, Tremona Road, Southampton, United Kingdom; 5 Department of Hepatology, Division of Medicine, Imperial College, London, United Kingdom; Kunming Institute of Zoology, Chinese Academy of Sciences, China

## Abstract

**Background:**

Genetic variation in the *IL28B* gene has been strongly associated with treatment outcomes, spontaneous clearance and progression of the hepatitis C virus infection (HCV). The aim of the present study was to investigate the role of polymorphisms at this locus with progression and outcome of HCV infection in a Moroccan population.

**Methods:**

We analyzed a cohort of 438 individuals among them 232 patients with persistent HCV infection, of whom 115 patients had mild chronic hepatitis and 117 had advanced liver disease (cirrhosis and hepatocellular carcinoma), 68 individuals who had naturally cleared HCV and 138 healthy subjects. The *IL28B* SNPs rs12979860 and rs8099917 were genotyped using a TaqMan 5′ allelic discrimination assay.

**Results:**

The protective rs12979860-C and rs8099917-T alleles were more common in subjects with spontaneous clearance (77.9% vs 55.2%; p = 0.00001 and 95.6% vs 83.2%; p = 0.0025, respectively). Individuals with clearance were 4.69 (95% CI, 1.99–11.07) times more likely to have the C/C genotype for rs12979860 polymorphism (p = 0.0017) and 3.55 (95% CI, 0.19–66.89) times more likely to have the T/T genotype at rs8099917. Patients with advanced liver disease carried the rs12979860-T/T genotype more frequently than patients with mild chronic hepatitis C (OR = 1.89; 95% CI, 0.99–3.61; p = 0.0532) and this risk was even more pronounced when we compared them with healthy controls (OR = 4.27; 95% CI, 2.08–8.76; p = 0.0005). The rs8099917-G allele was also associated with advanced liver disease (OR = 2.34; 95% CI, 1.40–3.93; p = 0.0100).

**Conclusions:**

In the Moroccan population, polymorphisms near the *IL28B* gene play a role both in spontaneous clearance and progression of HCV infection.

## Introduction

Infection with hepatitis C virus (HCV) is a worldwide health problem, with more than 170 million individuals infected. This infection results in a chronic active hepatitis in more than 80% of the infected patients, of whom 20–30% develop progressive cirrhosis and hepatocellular carcinoma (HCC), conversely only about 10–20% of infected people spontaneously eliminate the virus [1]. In Morocco, the prevalence of anti-HCV antibody in the general population is estimated to be 1.1% [2]. More than 70% of HCV infections in Morocco are genotype 1b, which is the most common genotype in Western North Africa [3]. Therapy with pegintereron-alpha (PegIFN-α) and ribavirin (RVB) is successful in only about 50% of patients infected with genotype 1 [4]. Viral and host factors have been associated with the differences in HCV clearance or persistence, and previous studies have demonstrated that a strong host immune response against HCV favours viral clearance [5], [6]. Ethnic differences in the frequency of virus elimination suggest an involvement of host genetic variation in spontaneous clearance [7]. Four independent genome-wide association studies (GWAS) have recently identified several single nucleotide polymorphisms (SNPs) around the interleukin-28B (*IL28B)* gene, located on chromosome 19q13, coding for IFN-λ3, that are strongly associated with treatment outcome and spontaneous HCV clearance [8], [9], [10], [11]. The two strongest genetic predictors for spontaneous HCV and treatment clearance were SNPs rs12979860 and rs8099917 [8], [9], [11]. The rs12979860 SNP lies 3 kb upstream of the *IL28B* gene whereas rs8099917 is located 8.9 kb from the IFNλ3-encoding transcript 3′ end in the intergenic region between IFNλ2 and IFNλ3. These polymorphisms exhibit substantial ethnic diversity in their frequency [8], [9], [11].

Because of its particular geographical location, between the Mediterranean sea, the Atlantic Ocean, and the Sahara desert, Moroccan populations has a dual Berberic and Arabic ethnicity. They show limited genetic diversity as the Arabs and Berbers are closely related [12]. Although the association between *IL28B* polymorphisms and the outcome of HCV infection is well recognised, they have not been studied in Moroccan or in other Maghreb populations. Furthermore there are relatively few studies that have investigated whether this genetic variant also influence the progression of chronic HCV infection [13], [14], [15], [16], [17].

In this study, we tested the hypothesis that the SNPs rs12979860 and rs8099917, and their combined effect, are associated with spontaneous clearance and progression of HCV infection in a Moroccan population, in addition to treatment response. To test this hypothesis, we used a prospectively followed up Moroccan cohort that was well characterized in terms of the natural outcome of HCV infection, the stage of disease and treatment response.

## Materials and Methods

### Patients

To participate in the study, written informed consent for genetic testing including *IL28B* polymorphisms and other potential markers was obtained from all individuals. Each participant was interviewed and completed a structured questionnaire to elicit demographic data and selected risk factors. The study protocol was evaluated and approved by the ethics Committee of the Faculty of Medicine of Casablanca and the study was conducted in accordance with the ethical guidelines of the 1975 Declaration of Helsinki as reflected in a priori approval by the institution’s human research committee. Both plasma and peripheral blood mononuclear cells (PBMCs) were stored for all patients. A cohort consisting of 438 Moroccan individuals were enrolled in this study at the Medical Center of Biology at the Pasteur Institute of Morocco and Service of Medicine B CHU Ibn Rochd hospital, Casablanca from January 2010 to October 2012. Two hundred and thirty-two subjects had persistent HCV infection. One hundred and eight were male and 124 female. All were persistently positive for anti-hepatitis C virus (anti-HCV) antibodies and HCV RNA by a quantitative reverse transcriptase-polymerase chain reaction (qRT-PCR) for at least six months. Patients were stratified into two groups according to fibrosis stage as determined by histology. One hundred and fifteen patients had mild chronic hepatitis C (mCHC) (patients with F0 and F1) and 117 patients had advanced liver disease (AdLD) (57 cirrhosis without HCC and 60 cirrhosis with HCC). Sixty-eight individuals (male cases, n = 21 and female cases, n = 47) had spontaneously resolved HCV infection. All were positive for HCV-specific antibodies and negative for HCV RNA in patient’s sera by qRT-PCR from at least two measurements more than 6 months apart. Controls consisted of 138 unrelated healthy subjects of mixed Berberic and Arabic ethnicity, who were negative for viral hepatitis markers and had normal serum levels of alanine aminotransferase (ALT) and aspartate aminotransferase (AST). All patients were HBsAg and HIV negative. Serological markers for HBsAg, anti-HCV and anti-HIV were tested with commercially available kits (Axsym, Abbott Diagnostics, Wiesbaden-Delkenheim, Germany and Genscreen Ag/Ab HIV Ultra, Biorad, Marnes La Coquette, France). Plasma HCV-RNA was measured using COBAS AmpliPrep/COBAS TaqMan (Roche Diagnostics, Germany). Hepatitis C virus genotypes were determined by sequencing as described previously [3].

### Isolation of Genomic DNA and SNPs Genotyping

Genomic DNA was isolated from PBMC using QIAamp DNA Blood Mini Kit (Qiagen, Valencia, CA) according to the manufacturer’s instructions. Genomic DNA concentration was assessed using a NanoVue plus spectrophotometer (GE Healthcare, US). Genotyping for the SNP rs12979860 was performed by means of a TaqMan 5′ allelic discrimination assay. The primers used were forward 5′-TGCCTGTCGTGTACTGAACCA-3′ and reverse 5′-GAGCGCGGAGTGCAATTC-3′. The sequences of the Taqman probes were TGGTTCGCGCCTTC and CTGGTTCACGCCTTC. The probes were labelled with the fluorescent dyes VIC and FAM, respectively. The PCR reaction was carried out in a total volume of 25µl, containing 20 ng of genomic DNA, with the following amplification protocol: pre-incubation at 50°C for 2 min and then 95°C for 10 min, followed by 40 cycles of denaturation at 95°C for 15 s, and annealing/extension at 60°C for 1 min. The genotype of each sample was attributed by the SDS 1.1 software for allelic discrimination (ABI7000, Applied Biosystems, Foster City, CA, USA). Most of the genotypes were confirmed by single base sequencing of PCR products using primers Forward 5′-GCTTATCGCATACGGCTAGGC-3′ and reverse 5′-TTCCCATACACCCGTTCCTGT-3′, PCR was carried out in a final volume of 25µl, containing 50 ng of genomic DNA, 20 pmol/µl of each primer, 0.5 unit of GoTaq DNA polymerase (Promega, France), 200µM of each dNTP and 1.5 mM MgCl_2_. The resulting 367-bp PCR product was purified using the Exonuclease I/Shrimp Alkaline Phosphatase (GE Healthcare, US) and sequenced using BigDye Terminator version 3.1 Cycle Sequencing kit (Applied Biosystems, Foster City, CA, USA) and an ABI PRISM 3130 DNA automated sequencer (Applied Biosystems, Foster City, CA, USA). Sequencing data were analyzed using SeqScape v2.5 software (Applied Biosystems, Foster City, CA, USA). The SNP rs8099917 was genotyped using a predesigned TaqMan SNP Genotyping Assay (Applied Biosystems; assay ID C_11710096_10). For 20% of the samples, genotyping was repeated for quality control. The results had 100% concordance.

### Statistical Analysis

Continuous variables are presented as mean ± standard deviation or median (range) while categorical variables are expressed as frequencies (%). Differences between continuous variables were analyzed using the Mann-Whitney U test, whilst those between categorical variables were evaluated using the Pearson chi-square test or Fisher’s exact test. Departures from Hardy-Weinberg equilibrium were determined by comparing the observed genotype frequencies with expected genotype frequencies in all groups calculated using observed allele frequencies by chi-square G test ‘‘Goodness of Fit’’ with 1 degree of freedom using website http://ihg2.helmholtz-muenchen.de/ihg/snps.html. The existence of differences in allelic and genotypic frequencies between different groups was assessed by means of chi-square test for linear trend when appropriate and calculating the odds ratio (OR) with the 95% confidence intervals (CI). Multiple logistic regression models were used to asses whether *IL28B* rs12979860 C/T and *IL28B* rs8099917 T/G polymorphisms could be considered a predictor of spontaneous resolution and the risk of advanced liver disease. A P value of <0.05 was used as the criterion for statistical significance. All the statistical analyses were performed using Statistical Package for Social Sciences (SPSS) program (version 10.0, SPSS Inc., Chicago, IL, USA).

## Results

The demographic data, biochemical features, viral load, viral genotype, and clinical features of our cohort groups are summarized in Table 1. Two hundred and thirty-two patients with HCV persistent infection with a mean age of 63.66±12.26 years [range, 20–90 years], 68 individuals with spontaneous resolution with a mean age of 57.77±15.64 years [range, 17–83 years] and 138 normal controls with a mean age of 56.26±10.57 years [range, 25–85 years] were enrolled in this study. There were no statistically significant differences in the distributions of sex and age between all groups (p>0.05). The mean serum ALT and AST levels was significantly higher in the persistently infected group compared with resolved and control groups (p<0.05). With regard to the viral genotypes, 70% of the patients were infected with viral genotype 1 and 30% of patients with genotype 2 (Table 1). This result is consistent with a recently published report showing the predominance of genotype 1 in Moroccan patients [3].

**Table 1:** Demographic and clinical characteristics of the study subjects.

	Persistent infection (n = 232)	Spontaneous clearance (n = 68)	Healthy controls (N = 138)
Mean age ± SD, y	63.66±12.26	57.77±15.64	56.26±10.57
Gender (%)			
Male	48	31	48
Female	52	69	52
Alanine aminotransferase (IU/L)	80.38±49.41	31.03±14.90	26.53±9.41
Aspartate aminotransferase (IU/L)	75.59±50.81	29.03±17.08	25.21±9.37
Mean Bilirubin (µmol/L)	26.77±10.20	10.07±2.70	
Mean creatinin (mmol/l)	112.57±105.15	77.28±26.23	
Median Viral Load (IU/ml)	835817 [2030–2.8.10^8^]		
Viral genotypes (%)			
Genotype 1	70		
Genotype 2	30		
mCHC‡	115		
Advanced Liver Disease (AdLD)*	117		

‡ Patients with mild chronic hepatitis C.

* including patient with liver cirrhosis and hepatocellular carcinoma.

To estimate the frequencies of the *IL28B* genotypes in Morocco population, the SNPs rs12979860 and rs8099917 were genotyped in healthy controls (Table 2). Overall, the genotype distribution at the rs12979860-C/T locus was as follows: 64 (46%) individuals were C/C homozygous, 59 (43%) were heterozygous and 15 (11%) were T/T homozygotes. At the SNP rs8099917-T/G, the distribution was T/T 80%, T/G 20%, G/G 0%. The allele frequency at the rs12979860 SNP was as follows: 68% for the C allele (95% confidence interval (CI) ±3%) and 90% ±1.69% for the T allele for the rs8099917 SNP. The genotype distributions at both SNPs were in Hardy-Weinberg equilibrium in the healthy control (for the SNP rs12979860, p = 0.845 and for the SNP rs8099917, p = 0.363).

**Table 2:** Effect of *IL28B* polymorphisms on the outcomes of HCV infection.

	Healthy controls	Subjects with persistence	Subjects with spontaneous clearance	Subjects with persistence vs. Subjects with spontaneous clearance	OR (95% CI)	P-value¥
**rs12979860**	n = 276	n = 464	n = 136			
C allele	0.680±0.03	0.552±0.026	0.779±0.040	C vs T	2.87 (1.84–4.48)	0.00001
T allele	0.320±0.03	0.448±0.026	0.221±0.040			
	n = 138	n = 232	n = 68			
C/C	64 (46.4%)	89 (38.4%)	45 (66.2%)	C/C vs T/T	4.69 (1.99–11.07	0.0017
C/T	59 (42.7%)	78 (33.6%)	16 (23.5%)	C/C vs C/T	2.46 (1.29–4.70)	0.0543
T/T	15 (10.9%)	65 (28%)	7 (10.3%)	C/C vs C/T and T/T	3.14 (1.78–5.55	0.0004
**rs8099917**	n = 276	n = 464	n = 136			
T allele	0.902±0.017	0.832±0.017	0.956±0.017	T vs G	4.38 (1.86–10.28)	0.0025
G allele	0.098±0.017	0.168±0.017	0.044±0.017			
	n = 138	n = 232	n = 68			
T/T	111 (80.4%)	158 (68.1%)	62 (91.2%)	T/T vs T/G	4.58 (1.89–11.08)	0.0029
T/G	27 (19.6%)	70 (30.2%)	6 (8.8%)	T/T vs G/G	3.55 (0.19–66.89)	0.2110
G/G	0 (0%)	4 (1.7%)	0 (0%)	T/T vs T/G and G/G	4.84 (2.00–11.69)	0.0017

¥ Bonferroni correction was applied.

In order to analyze the impact of polymorphisms of *IL28B* gene in the Moroccan population, we genotyped rs12979860 (C/T) and rs8099917 (T/G) polymorphisms in patients with persistent HCV infection and individuals who had spontaneously cleared the virus (Table 2). Subjects with the rs12979860 C/C genotype cleared HCV infection more often those with the rs12979860-T/T genotype (OR = 4.69; 95% CI, 1.99–11.07; p = 0.0017) or the rs12979860-CT genotype (OR = 2.46 (95% CI, 1.29–4.70; p = 0.0543) (Table 2). Interestingly, when individuals were stratified according to their rs12979860 genotypes (C/C vs. C/T+T/T), the C/C genotype was overrepresented in the resolved group (66.2%) compared to the persistent HCV infection group (38.4%) (p = 3×10^−5^). The rs12979860 C/C genotype was associated with resolution of HCV in both males and females. These results were confirmed by analysis of the linked SNP rs8099917 (Table 2). Genotype distributions were in Hardy-Weinberg equilibrium in both groups (p = 0.345 and 1.00, respectively). 158 of 232 patients (68.1%) with persistent infection were homozygous for T/T, 70 (30.2%) were heterozygous (T/G) and 4 (1.7%) homozygous (G/G), reflecting a T-allele frequency of 83.2%. In individuals with spontaneous clearance the T/T genotype was found in 62 (91.2%) individuals and the G/T genotype in 6 (8.8%). The frequency of the T allele (95.6%) was significantly greater among individuals with HCV clearance as compared to those with persistent infection (83.2%) (p = 0.0025) (Table 2). The *IL28B* T/T genotype was found less frequently in the persistent group compared to spontaneous resolvers (68.1% vs. 91.2%, p = 4×10^−5^). Interestingly, when patients with persistent HCV infection were stratified by their viral genotypes, the frequencies of the C/T+T/T genotypes in patients infected with genotype 1 (67.3%) was more prevalent than in patients with genotype 2 (53.5%) (p = 0.0432). Whereas, for the SNP rs8099917, the frequency of G/T+G/G in patients with genotype 1 was 39.8% which was higher than in those infected with genotype 2 (27.9%) (p = 0.0641). This is consistent with a differential effect of the IL-28B polymorphisms on the outcome of genotype 1 versus genotype 2 HCV infection.

To test the association between the SNP rs12979860 and the progression of HCV disease, we genotyped this polymorphism in 117 patients with advanced liver disease (patients with cirrhosis and/or HCC) and compared them with 115 patients with mCHC (Table 3). The frequency of the T/T genotype was significantly overrepresented in advanced liver disease patients compared with mCHC (p = 0.0366) a trend even more pronounced when we compared these frequencies with those of healthy controls (11%, p = 2×10^−5^, Table 3). The frequency of the rs12979860-T allele in advanced liver disease (50%) was also significantly higher than in patients with mCHC (39.6%) (p = 0.0238). In multivariate logistic regression analysis the association of the rs12979860-T/T genotype with advanced liver disease compared with mCHC revealed an OR of 1.89 (CI, 0.99–3.61; p = 0.0532) after adjustment for age, sex and viral genotype.

**Table 3:** Effect of *IL28B* polymorphisms on the progression of HCV infection.

	Healthycontrols(N = 138)	mCHC group(n = 115)	HCV-AdLDgroup(n = 117)	mCHC vs. AdLDOR (95% CI)	P-value¥	Healthy controlsvs. AdLDOR (95% CI)	P-value¥
Mean age ± SD	56.26±10.57	60.58±14.21	66.53±9.26		0.0039		
Male/Female	66/72	44/71	64/53		0.0130		
Genotype 1	-	65%	72%		0.4360		
Genotype 2	-	35%	28%				
**rs12979860**							
C/C	64 (46.4%)	51 (44.3%)	38 (32.5%)	1.00		1.00	
C/T	59 (42.7%)	37 (32.2%)	41 (35%)	1.49 (0.81–2.74)	0.2025	1.17 (0.66–2.06)	0.5855
T/T	15 (10.9%)	27 (23.5%)	38 (32.5%)	1.89 (0.99–3.61)	0.0532	4.27 (2.08–8.76)	0.0005
C allele	0.680±0.03	0.604±0.037	0.500±0.037	1.00		1.00	
T allele	0.320±0.03	0.396±0.037	0.500±0.037	1.53 (1.06–2.21)	0.0238	2.10 (1.47–3.01)	0.0005
**rs8099917**							
T/T	111 (80.4%)	91 (79.1%)	68 (58.1%)	1.00		1.00	
T/G	27 (19.6%)	23 (20%)	46 (39.3%)	2.68 (1.48-4.83)	0.0091	2.78 (1.58–4.88)	0.0029
G/G	0 (0%)	1 (0.9%)	3 (2.6%)	4.00 (0.41–39.44)	0.1991	11.94 (0.58–223.97)	0.2898
T allele	0.902±0.017	0.891±0.020	0.778±0.025	1.00		1.00	
G allele	0.098±0.017	0.109±0.020	0.222±0.025	2.34 (1.40–3.93)	0.0100	2.63 (1.59–4.36)	0.0011

* Adjusted for age, gender and viral genotype.

¥ Bonferroni correction was applied.

We found a similar effect for the SNP rs8099917. In multivariate logistic regression analysis after adjustment for age and sex we found that the genotypes T/G+G/G were higher in those with advanced liver disease as compared to mCHC (42% versus 21%, respectively) (p = 0.0002) (Table 3). Taking the T/T genotype as the reference, the OR and 95% CI for the T/G and G/G genotypes presence in the advanced versus mild groups were 2.68±1.48−4.83 and 4.00±0.41−39.44 respectively (Table 3).

Data for clearance following PegIFN-α plus RVB treatment were available in 41 patients. For the SNP rs12979860, there is a statistical difference (p = 0.035) was observed for the distribution of the genotype C/C [75%, n = 6] and genotypes C/T and T/T [25%, n = 2] between responders and non-responders (genotype C/C [33%, n = 11] and genotypes C/T and T/T [67%, n = 22], Fisher’s exact test). Furthermore the rs8099917 polymorphism was associated with SVR. In responders the frequency of patients with T/T genotype (80%) was higher than in non-responders (60%) (OR = 0.46, 95% CI 0.22–1.09; p = 0.019).

The analysis of the combination of SNPs rs12979860 with rs8099917 showed that patients with persistent infection were 8.27 (95% CI, 1.90–36.11; p = 0.0011) times more likely to have the rs12979860-T/T/rs8099917-T/G genotypes (13.80% vs 2.94% in resolved group) and 4.68 (95% CI, 0.25–88.85) fold to have rs12979860-TT/rs8099917-GG genotypes (1.72% vs none in resolved group) but did not reach the significance (p = 0.155) when compared to rs12979860-C/C/rs8099917-T/T carriers.

Therefore, we hypothesized that an increased risk of progression towards advanced liver disease could be detected when unfavourable alleles are combined in a given patient. Consequently, the SNP-SNP combination of rs12979860 with rs8099917 was examined. We found that the rs12979860-T/T/rs8099917-T/G genotype was present in 19.6% of the advanced group versus 6.9% in mCHC (p = 0.0035), conferring an OR of 3.70 (95% CI, 1.49–9.20, p = 0.0035) for advanced liver disease. Additionally the frequency for patients with rs12979860-T/T/rs8099917-G/G genotypes was 2.6% in the advanced group vs 0.9% in mCHC conferring a 3.87-fold increased risk for advanced liver disease compared to rs12979860-C/C/rs8099917-T/T carriers.

## Discussion

The ability of the virus to persist within a host’s attributed to its efficient ability to evade the adaptive and innate components of the host’s immune system. In addition, a small number of specific host polymorphisms have been correlated with spontaneous HCV clearance [18]. The SNPs near the *IL28B* gene on chromosome 19 coding for type III IFN-λ3 have recently been reported to be associated with clearance and treatment response in HCV [8], [19]. First, we analyzed the *IL28B* polymorphisms in the 138 healthy subjects. The rs12979860-C allele frequency observed in the Moroccan population (68%) was similar to that reported in the Egyptian population (67%) [20] and in the Italian population (68%) [13], [21] but was significantly higher than those observed within the sub-Saharan African populations (23.1–54.8%) [19]. However, the C allele frequency remains much lower than the highest frequencies found in Eastern Asian (91–100%) and in Oceanian populations (100%) [19]. The frequency of the favourable T allele of the rs8099917 SNPwas 90% amongst Moroccan subjects, which is similar to frequencies reported in African (93%) and European (83%) populations but was higher than those observed in a population with Mexican ancestry (69%) [22]. Thus ethnic differences in the *IL28B* gene polymorphisms may explain, at least in part, the different outcomes rates of HCV infection in different ethnic groups. For this reason, we examined the genetic variation in the *IL28B* gene and the natural clearance of HCV infection in the Moroccan population. Our results demonstrate that the rs12979860- C/C genotype is strongly associated with spontaneous eradication of the virus in the Maghreban populations. Our results are consistent with previous studies of the association of rs12979860-C/C genotype with spontaneous clearance. We found a relationship with the highest clearance rate among C/C Moroccan subjects (66.2%), an intermediate clearance rate among heterozygous individuals (23.5%), and the lowest rate in the T/T homozygous subjects (10.3%). For spontaneous HCV clearance, Thomas et al. reported that the *IL28B* rs12979860-C/T polymorphism predicted the rate of spontaneous clearance of HCV, with the frequency of spontaneous clearance being 53% in patients with the C/C genotype versus 23% of patients with the T/T genotype [19]. Several larger studies reported that the rs12979860-C/C genotype was associated with spontaneous eradication of the virus [20], [23], [24], [25], [26], [27] (Table S1). Moreover, Langhans and coworkers demonstrated that high IFN-λ serum levels were prevalent in carriers of the rs12979860-C allele and associated with a favourable outcome of HCV infection confirming the important antiviral properties of type III interferons (IFNs) [28].

The influence of *IL28B* polymorphisms on the severity and progression of liver disease remains unclear with controversial results [13], [14], [15], [16], [29], [30]. This emphasizes the necessity to replicate these studies in ethnically diverse populations. In our study, the frequency of patients with the rs12979860-T/T genotype differ between mCHC and HCV-AdLD groups (Table 3), suggesting that this polymorphism may play a role in the progression of HCV among Moroccan patients. Actually, in patients with advanced liver disease the frequency of the T allele was 50%, compared to 60.4% of patients with mild liver disease. Carriage of the T allele was found to be an independent predictor of progression to an advanced stage, which is consistent with the reports in other populations [13], [16], [29], [31]. The rs12979860-T/T genotype was previously been associated with a four-fold increase in risk for HCC development in Italian patients, particularly among patients infected with HCV [13]. Recently, Clark and colleagues found that the rs12979860-T/T genotype was associated with fibrosis in patients with chronic hepatitis C [32]. Moreover, several *in vitro* studies and animal models demonstrated that activation of type III IFN induces apoptosis and also that this cytokine possesses anti-tumour activities [33]. Thus, the SNP rs12979860 may serve as an important predictive biomarker of liver disease and offers new insights into the biological pathways involved in liver cirrhosis and carcinogenesis. In addition, it was reported that the rs8099917-T/T genotype was associated with spontaneous clearance of HCV (Table S1) and inflammatory activity and fibrosis in patients with chronic hepatitis C in Japan. Overall our results confirm this positive association between rs8099917 polymorphism and progression of HCV infection [11], [34]. Overall, elucidating the mechanism by which the *IL28B* polymorphisms affects the progression to cirrhosis and HCC remains an important future challenge.

In Morocco, HCV genotype 1 is more prevalent than genotype 2. In addition, the SVR rate for individuals infected with genotype 1, is low (40–50%) and requires 12 months of therapy. Results from our small cohort showed that patients who achieved SVR had a higher frequency of the rs12979860-C/C genotype (75%) compared to those carrying the T/T genotype (12.5%).

Finally, the combination of the two polymorphisms is associated with significantly increased persistence and linked to progression towards advanced stages of HCV-associated disease. Individuals with rs12979860-T/T genotype and T/G or G/G of rs8099917 have an 8.27 and 4.68-fold-risk to evolve towards chronicity respectively, and a 3.70 and 3.87 increase in risk to be affected with an advanced liver disease.

We are, however, fully aware that the limitation of the current study is its relatively small size and, further studies warrant the recruitment of a larger cohort with analysis of the above mentioned, and additional polymorphisms and haplotypes of the *IL28B* gene. These will help to clarify their role in HCV clearance and may provide a mechanism for understanding the relationship between IL28B and HCV infection.

In summary, our results are the first providing information about the impact of IL28B polymorphisms on hepatitis C outcomes and progression in the Maghrebian region. We found that rs12979860-C/C and rs8099917-T/T genotypes showed a strong association with resolution of HCV infection. Altogether, our result support that *IL28B* polymorphisms seems to be involved in the progression of HCV infection to cirrhosis and hepatocellular carcinoma. This finding adds evidence to the hypothesis that the host’s immunogenetic background modulates the progression of liver disease in chronic hepatitis C in multiple and ethnically diverse populations.

## Supporting Information

Table S1 Main results of IL28B polymorphisms and spontaneous clearance of HCV infection.
(DOCX)
