# Supplementary material for: Genetic Variation in the Interleukin-28B Gene Is Associated with Spontaneous Clearance and Progression of Hepatitis C Virus in Moroccan Patients
Source: PLoS One. 2013 Jan 24;8(1):e54793. doi: 10.1371/journal.pone.0054793 (PMC3554614; doi:10.1371/journal.pone.0054793)
Supplement: Table S1 — Main results of IL28B polymorphisms and spontaneous clearance of HCV infection. (DOCX) [file pone.0054793.s001.docx]

**Table S1**: Main results of IL28B polymorphisms and spontaneous clearance of HCV infection

| IL28B Polymorphisms | Ancestry | HCV Genotypes | Persistence No. | Clearance No. | OR (95% CI) | P-Value | Reference |
| --- | --- | --- | --- | --- | --- | --- | --- |
| **rs12979860^†^** |  |  |  |  |  |  |  |
|  | European, African (US) | 1,2 | 620 | 388 | 0.29 (0.18-0.47) | 4x10^-7^ | [19] |
|  | Caucasian (Egypt) | 4 | 82 | 80 | 3.8 (1.3-11.5) | 0.0126 | [20] |
|  | Caucasian (Egypt) | 4 | 131 | 130 | 2.84 (1.87-4.30) | 1.6x10^-7^ | [26] |
|  | African (US) | 1, 2 | 348 | 111 | 3.12 | < 0.001 | [24] |
|  | European (US) | 1, 2, 3 | 392 | 157 | 4.67 | < 0.001 | [24] |
|  | Asian (China) | 1, 2 | 529 | 196 | 2.12 (1.01-4.42) | 0.04 | [23] |
|  | Caucasian (Spain) | 1, 2, 3, 4 | 284 | 69 | 0.32 (0.18-0.57) | 6.2x10^-5^ | [30] |
|  | Caucasian (Italy) | 1, 2, 3, 4 | 147 | 98 | 2.42 (1.44- 4.09) | 0.001 | [27] |
|  | Caucasian (Morocco) | 1, 2 | 232 | 68 | 4.69 (1.99-11.07) | 0.0017 | This study |
| **rs8099917^¥^** |  |  |  |  |  |  |  |
|  | Caucasian (Italy) | 1, 2, 3, 4 | 147 | 98 | 2.13 (1.22-3.73) | 0.008 | [27] |
|  | African, European, Asian (US) | 1, 2, 3 | 883 | 326 | 0.13 (0.03-0.56) | 0.006 | [24] |
|  | Asian (China) | 1, 2 | 160 | 80 | 15.3 (2.1-112.5) | 0.002 | [33] |
|  | Caucasian (Swiss) | 1, 2, 3, 4 | 1015 | 347 | 2.49 (1.64-3.79) | 1.96x10^-5^ | [11] |
|  | Caucasian (Egypt) | 4 | 131 | 130 | 2.61 (1.51-4.49) | 2.74x10^-4^ | [26] |
|  | Caucasian (Morocco) | 1,2 | 232 | 68 | 4.84 (2.00-11.69) | 0.0017 | This study |

^†^C/C vs C/T or T/T

^¥^T/T vs T/G or G/G
